# Fragility Score in Radiographic Axial Spondyloarthritis Assessed with Radiofrequency Echographic Multi-Spectrometry (REMS)

**DOI:** 10.3390/life16071121

**Published:** 2026-07-05

**Authors:** Elena Bischoff, Stoyanka Vladeva, Nikola Kirilov, Fabian Bischoff

**Affiliations:** 1 Department of Health Care, Faculty of Medicine, Trakia University, 6000 Stara Zagora, Bulgaria; 2 Rheumazentrum Ruhrgebiet Herne, Ruhr-Universität Bochum, 44649 Herne, Germany; 3 Department of Orthopedics and Traumatology, University Hospital UMBAL Dr. Georgi Stranski, Medical University of Pleven, 5803 Pleven, Bulgaria; 4 Rheumatology Practice Stara Zagora, 6000 Stara Zagora, Bulgaria

**Keywords:** radiographic axial spondyloarthritis, skeletal fragility, bone mineral density, fracture risk, Radiofrequency Echographic Multi-Spectrometry (REMS), Fragility Score, bone quality

## Abstract

Axial spondyloarthritis (axSpA) is a chronic inflammatory disease affecting the sacroiliac joints and spine and is associated with an increased risk of fractures due to persistent inflammation, reduced mobility and treatment-related factors. In radiographic axSpA (r-axSpA), assessment of bone mineral density (BMD) using dual-energy X-ray absorptiometry (DXA) may be limited by structural spinal changes. This cross-sectional study, conducted between March 2024 and June 2025, evaluated skeletal fragility in patients with r-axSpA using Radiofrequency Echographic Multi-Spectrometry (REMS)-derived Fragility Score (FS). Ninety patients with r-axSpA and sex-matched healthy controls underwent clinical assessment and REMS evaluation of lumbar spine and hip BMD, T-scores and spinal FS. Patients with r-axSpA had lower body mass index and higher rates of smoking, prior fractures and inflammatory markers compared with controls, while disease activity reflected a moderate burden. No significant differences in BMD or T-scores were observed between groups. However, FS was significantly higher in patients with r-axSpA (46.6 ± 15.4 vs. 31.2 ± 13.3, *p* = 0.004), corresponding to a higher fracture risk category, whereas correlations between FS and clinical parameters were not statistically significant. These findings suggest that REMS-derived FS may identify increased skeletal fragility in r-axSpA beyond conventional BMD measurements.

## 1. Introduction

Axial spondyloarthritis (axSPA) is a chronic inflammatory rheumatologic disease. It is characterized by inflammation of the sacroiliac joints and the spine, which can lead to structural damage and ultimately ankylosis. AxSPA comprises several disease entities, including psoriatic arthritis, reactive arthritis, ankylosing spondylitis and inflammatory bowel disease (IBD)-associated arthritis [1].

It is well established that autoimmune diseases within the rheumatic spectrum are associated with reduced bone mass [2,3]. Several underlying mechanisms have been identified. On the one hand, local inflammatory processes in the spine lead to bone loss [4,5]; on the other hand, autoinflammation results in the release of cytokines that negatively affect bone health [6,7]. Furthermore, individuals positive for the HLA-B27 surface antigen, which is known to be associated with axial spondyloarthritis [8,9], have been shown to have an increased prevalence of osteoporosis [10]. In patients with axial spondyloarthritis, progressive immobility over the disease course may additionally contribute to reduced bone mineral density [11]. In axial spondyloarthritis, glucocorticoids are also used as part of the therapeutic approach. It is well established that glucocorticoid therapy leads to a reduction in bone mineral density [12,13,14].

Given the increased susceptibility to impaired bone health and fractures in patients with r-axSpA, accurate assessment of bone mineral density and skeletal fragility is essential for timely diagnosis and appropriate clinical management. However, the structural skeletal changes characteristic of advanced disease present important challenges for conventional imaging techniques used to evaluate bone health.

Despite its widespread use, conventional dual-energy X-ray absorptiometry (DXA) has important limitations in patients with radiographic axial spondyloarthritis (r-axSpA). Structural spinal changes, including syndesmophyte formation, ligament ossification, and ankylosis may lead to falsely elevated lumbar spine bone mineral density measurements, potentially underestimating skeletal fragility in this patient population. Consequently, alternative methods capable of assessing bone health without being substantially influenced by these structural changes are of considerable clinical interest.

Radiofrequency Echographic Multi-Spectrometry (REMS) is a radiation-free, ultrasound-based technique for assessing bone mineral density at the lumbar spine and femoral neck, providing measurement sites comparable to those of DXA [15,16]. Previous studies have demonstrated good agreement between REMS and DXA for bone mineral density assessment [17,18]. Unlike conventional ultrasound imaging, REMS analyzes raw radiofrequency signals using automated signal-processing algorithms to estimate bone mineral density and bone quality. In addition to bone mineral density and T-score, REMS provides information on bone quality through the Fragility Score (FS), which may offer additional insight into skeletal fragility beyond bone quantity alone. By minimizing the influence of artifacts such as osteophytes and vascular calcifications, REMS has the potential to overcome some of the limitations of conventional DXA, particularly in patients with structural spinal changes.

The principle of REMS technology is based on the processing and analysis of reflected and absorbed ultrasound waves. The device analyzes transmitted and received ultrasound data across different spectra and derives bone mineral density from these signals. In addition, it provides information not only on bone mineral density but also on bone quality, summarized in the so-called Fragility Score (FS). This is a dimensionless score ranging from 0 to 100. The Fragility Score is age-adjusted and categorized into normal bone quality, reduced bone quality and low bone quality, with higher values indicating poorer bone quality. Together with the REMS-derived T-score, a fracture risk for major osteoporotic fractures is calculated, expressed per 1000 individuals. The FS-based fracture risk is classified into seven risk categories (R1–R7), where R1 indicates low risk and R7 high risk. Studies have already shown that, despite apparently normal bone mineral density, fracture risk may still be elevated [19].

In radiographic axSpA (r-axSpA), conventional bone mineral density (BMD) assessment by DXA may be confounded by syndesmophyte formation and spinal ossification.

The present study aimed to investigate cross-sectional skeletal status in patients with established r-axSpA using REMS-derived FS and to compare REMS-derived skeletal parameters between patients and healthy controls.

## 2. Materials and Methods

### 2.1. Study Design and Population

We conducted a cross-sectional observational study between March 2024 and June 2025, including patients with r-axSpA and a control group. Patients with r-axSpA were consecutively recruited from a rheumatology outpatient clinic. Controls were selected to achieve a comparable sex distribution and had no known inflammatory rheumatic disease.

The study was conducted in accordance with the Declaration of Helsinki and was approved by the Ethics Committee for Scientific Research of the Medical Faculty, Trakia University, Stara Zagora, Bulgaria (Protocol No. 26, dated 1 June 2023).

Patients aged ≥18 years were eligible for inclusion, provided that informed consent was obtained prior to participation. Participants were excluded if they did not provide informed consent, had a previously confirmed diagnosis of other autoimmune or autoinflammatory rheumatic diseases, had an active infection or malignancy at the time of assessment, were unable to complete study procedures or had cognitive or language barriers that interfered with data collection or interpretation of results.

### 2.2. Clinical and Demographic Assessment

Demographic data included age, sex and body mass index (BMI). In the r-axSpA group, disease-related characteristics such as disease duration and human leukocyte antigen B27 (HLA-B27) status were recorded.

Lifestyle and clinical risk factors, including smoking status, history of fragility fractures and postmenopausal status in women were documented for all participants. A fragility fracture was defined as a fracture that occurred from a low-energy trauma that would not normally cause a fracture in healthy bone, such as a fall from standing height.

### 2.3. Disease Activity and Functional Assessment

In patients with r-axSpA, disease activity was assessed using the Bath Ankylosing Spondylitis Disease Activity Index (BASDAI) and the Ankylosing Spondylitis Disease Activity Score based on C-reactive protein (ASDAS-CRP). Functional status was evaluated using the Bath Ankylosing Spondylitis Functional Index (BASFI), while structural mobility impairment was assessed using the Bath Ankylosing Spondylitis Metrology Index (BASMI).

### 2.4. Laboratory Assessment

Inflammatory markers included CRP and erythrocyte sedimentation rate (ESR), measured using standard laboratory methods. CRP was determined in serum and expressed in mg/L, while ESR was measured using the Westergren method and expressed in mm/h. All analyses were performed in a certified clinical laboratory according to standardized protocols under routine quality control procedures.

### 2.5. Treatment Assessment

Current pharmacological treatments were recorded, including nonsteroidal anti-inflammatory drugs (NSAIDs), conventional synthetic disease-modifying antirheumatic drugs (csDMARDs), biologic DMARDs (tumor necrosis factor alfa inhibitors and interleukin-17 inhibitors) and systemic glucocorticoids. Bone-health-related supplementation, including calcium and vitamin D, as well as anti-osteoporotic therapy was also documented.

### 2.6. Bone Assessment Using REMS

The operator performing the REMS examinations was blinded to the participants’ clinical diagnosis and group allocation throughout image acquisition and analysis. A dedicated echographic device EchoStudio (Echolight S.p.a., Lecce, Italy), equipped with a convex transducer operating at a central frequency of 3.5 MHz, was positioned transabdominally and over the hip region to visualize the regions of interest (ROI), following a standardized acquisition protocol. REMS measurements were performed according to the manufacturer’s standardized acquisition protocol. Once an appropriate anatomical B-mode ultrasound image was obtained, the REMS software (version 2.2.1) automatically identified the bone interfaces at the target site. During image acquisition, sequences of ultrasound frames were recorded (approximately 80 s for the lumbar spine and 40 s for the proximal femur), allowing automatic identification of the region of interest for diagnostic evaluation.

The REMS algorithm analyzes the raw backscattered radiofrequency ultrasound signals rather than relying solely on conventional B-mode images. Automated signal processing procedures identify and exclude artifact signals characterized by unexpected spectral features, including those generated by vascular calcifications, osteophytes, implanted metallic hardware, orthopedic cement, and other non-representative structures. Following artifact rejection, the software synthesizes the remaining cortical and trabecular bone signals into a patient-specific spectral profile, generating a unique frequency spectrum of the backscattered radiofrequency signals from the selected region of interest.

The patient-specific spectral profile is subsequently compared with validated reference spectral models matched for sex, age, skeletal site, and body mass index (BMI) using the manufacturer’s proprietary database. Based on this comparison, the REMS software automatically calculates bone mineral density (BMD), T-score, Z-score and Fragility Score (FS).

**Figure 1:**
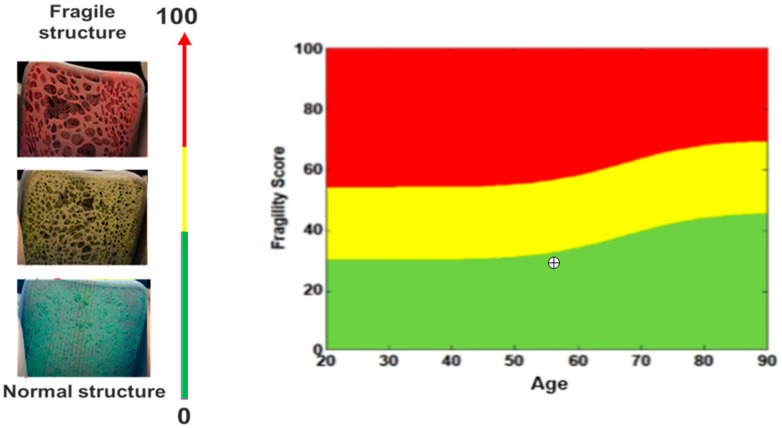
Graphical representation of the REMS-derived Fragility Score plotted against age on a color-coded reference chart. The patient’s Fragility Score is indicated by a dot with a cross in the center.

The FS is derived as a marker of skeletal fragility obtained from the REMS examination. This dimensionless parameter is calculated by comparing raw ultrasound radiofrequency spectral data with reference models of fragile and non-fragile bone. Lower FS values indicate preserved bone microarchitecture and lower estimated fracture risk, whereas higher values are associated with impaired microarchitectural integrity and increased fracture risk at the assessed site (Figure 1).

**Figure 2:**
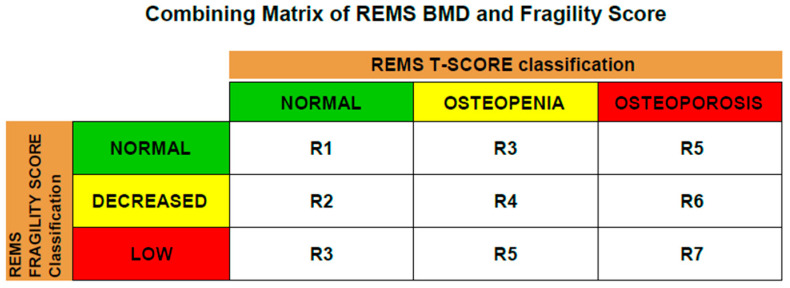
Interpretation of bone risk classification based on combined REMS T-score and Fragility Score (FS) values using the reference risk stratification table.

The physician can identify the risk class corresponding to the current patient combining measured REMS T score and FS values using the interpretation table (Figure 2).

### 2.7. Statistical Analysis

Statistical analyses were performed using IBM SPSS Statistics version 21.0 (IBM Corp., Armonk, NY, USA). Continuous variables were assessed for normality using visual inspection of histograms and the Shapiro–Wilk test. Normally distributed variables are presented as mean ± standard deviation, while categorical variables are expressed as frequencies and percentages.

Baseline characteristics between patients with r-axSpA and controls were compared using the independent samples *t*-test for continuous variables and the chi-square test or Fisher’s exact test, as appropriate for categorical variables. All tests were two-tailed and a *p*-value < 0.05 was considered statistically significant.

REMS-derived bone parameters, including bone mineral density (BMD), T-scores, and Fragility Score, were analyzed as continuous variables and compared between groups using independent samples *t*-tests.

Associations between clinical, laboratory and imaging variables and the REMS Fragility Score were assessed using Pearson correlation analysis. Correlation coefficients (r) and corresponding *p*-values were calculated to evaluate the strength and direction of relationships.

## 3. Results

Patients with r-axSpA (*n* = 90) were significantly younger than controls (46 ± 5 vs. 53 ± 4 years, *p* < 0.001). The proportion of female participants was comparable between groups (60% vs. 64%, *p* = 0.803). Body mass index was significantly lower in the r-axSpA group compared with controls (26.1 ± 3.5 vs. 31.5 ± 5.9 kg/m^2^, *p* < 0.001).

Disease-related variables were present only in the r-axSpA cohort with a mean disease duration of 7.5 ± 4.0 years and HLA-B27 positivity observed in 84% of patients. Lifestyle and clinical risk factors differed significantly between groups: current smoking was more frequent in r-axSpA patients (60% vs. 30%, *p* < 0.001), and previous fracture history was also higher (40% vs. 20%, *p* = 0.01). In contrast, postmenopausal status was more common in controls (80% vs. 44%, *p* = 0.01).

Inflammatory markers were markedly elevated in the r-axSpA group, with higher CRP (10 ± 12 vs. 2 ± 1.5 mg/L, *p* < 0.001) and ESR (25 ± 18 vs. 8 ± 6 mm/h, *p* < 0.001), consistent with active systemic inflammation. Disease activity and functional indices in the r-axSpA cohort indicated moderate disease burden, with BASDAI 4.8 ± 2.0, ASDAS-CRP 3.0 ± 1.0, BASFI 4.2 ± 2.1, and BASMI 3.5 ± 1.8.

Overall, the r-axSpA group was characterized by a higher inflammatory burden, greater smoking prevalence and increased fracture history compared with controls, despite a lower BMI (Table 1).

A multivariable linear regression analysis was performed to determine whether the association between r-axSpA and FS was independent of potential confounding factors, including age, sex, BMI, smoking status, previous fracture, postmenopausal status, CRP and ESR. The regression model was statistically significant overall (R^2^ = 0.59, adjusted R^2^ = 0.57, F = 29.8, *p* < 0.001), indicating that approximately 59% of the variability in FS was explained by the variables included in the model.

The study group (r-axSpA vs. control) remained the strongest independent predictor of FS (β = 0.39, 95% CI: 0.21–0.57, *p* < 0.001). Age was also independently associated with FS (β = 0.011, *p* = 0.006), confirming that FS increases with advancing age. In contrast, BMI was not an independent predictor of FS (β = 0.004, *p* = 0.576). Although BMI differed significantly between the groups in the univariate analysis, it did not contribute independently to FS after adjustment for the other variables. This suggests that the higher BMI observed in the control group did not explain the difference in FS scores. Similarly, female sex was not significantly associated with FS (*p* = 0.705), indicating that sex did not independently influence the outcome after controlling for the other covariates.

Among the clinical risk factors, current smoking was positively associated with FS (*p* = 0.032), suggesting a modest independent effect after adjustment. Previous fracture was also independently associated with higher FS scores (*p* = 0.020). Regarding inflammatory markers, CRP remained a significant independent predictor (*p* = 0.002), demonstrating that higher systemic inflammation was associated with increased FS scores. ESR also showed an independent but weaker association (*p* = 0.018). Postmenopausal status was not independently associated with FS (*p* = 0.446) after adjustment. This likely reflects the fact that much of its effect is captured by age, as menopausal status and age are strongly correlated (Table 2).

Overall, NSAID use was the most frequently reported therapy, with 68 patients (75%) receiving NSAIDs. A substantial proportion of patients were treated with bDMARDs, including TNF inhibitors in 36 patients (40%) and IL-17 inhibitors in 9 patients (10%). Conventional synthetic DMARDs were used in 28 patients (31%), while systemic glucocorticoid therapy was reported in 14 patients (15%).

Regarding bone-health-related supplementation, half of the cohort (45 patients, 50%) received calcium supplementation and a higher proportion (59 patients, 65%) were on vitamin D supplementation. Anti-osteoporotic pharmacological treatment was prescribed in 14 patients (15%).

Overall, the data reflect a treatment profile dominated by NSAIDs and biologic therapies, alongside frequent use of vitamin D and calcium supplementation, while targeted anti-osteoporotic therapy was comparatively less common (Table 3).

Overall, REMS-based BMD and T-score measurements showed no statistically significant differences between groups at any skeletal site. Lumbar spine REMS-based BMD was similar in r-axSpA patients and controls (0.810 vs. 0.821 g/cm^2^, *p* = 0.6), with corresponding T-scores indicating mild osteopenia in both groups (−1.4 vs. −1.3, *p* = 0.8). Comparable findings were observed at the femoral neck (REMS-based BMD: 0.735 vs. 0.760 g/cm^2^, *p* = 0.3; REMS-based T-score: −1.5 vs. −1.3, *p* = 0.6) and total hip (REMS-based BMD: 0.830 vs. 0.850 g/cm^2^, *p* = 0.2; REMS-based T-score: −1.1 vs. −0.9, *p* = 0.6).

In contrast, the REMS-derived Fragility Score was significantly higher in the r-axSpA group compared with controls (46.6 ± 15.4 vs. 31.2 ± 13.3, *p* = 0.004), suggesting increased bone fragility despite similar REMS-based BMD values. Correspondingly, patients with r-axSpA were classified into a higher risk category (R5) compared with controls (R4).

Overall, while standard REMS-based BMD and REMS-based T-score values did not differ between groups, REMS-based FS identified a significantly higher skeletal fragility burden in patients with r-axSpA (Table 4).

**Figure 3:**
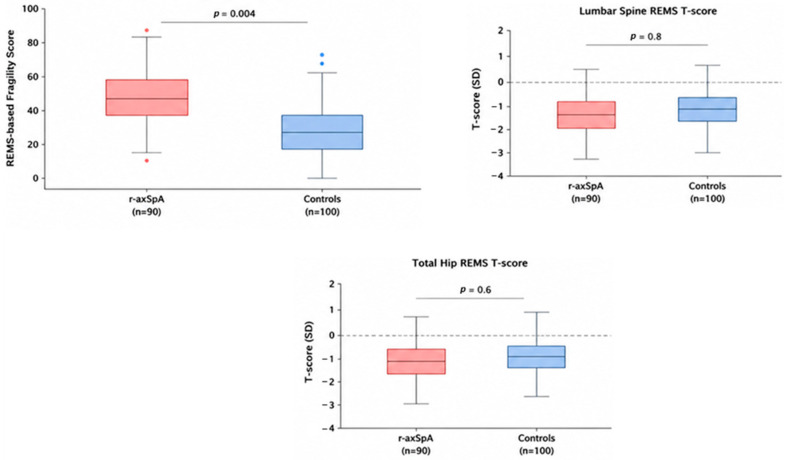
Box plots illustrating the distribution of FS scores and T-scores in patients with axSpA and healthy controls, highlighting the differences between the two groups. The dashed horizontal line represents the reference value of T-score = 0.

To facilitate a clearer comparison of the distributions of FS scores and T-scores between patients with axSpA and healthy controls, the data are presented as box plots. This visualization better illustrates the differences between the two groups by displaying the median, interquartile range, overall data spread, and potential outliers (Figure 3).

Overall, correlation coefficients of moderate magnitude were observed for several parameters, including BASMI (r = 0.48), BASFI (r = 0.42), age (r = 0.40), ASDAS-CRP (r = 0.38), disease duration (r = 0.32), lumbar spine REMS-based BMD (r = 0.32), CRP (r = 0.28), and BASDAI (r = 0.25). However, none of these correlations reached statistical significance (all *p* > 0.05), although BASMI approached statistical significance (*p* = 0.06). Therefore, no statistically significant associations were identified between the outcome measure and the evaluated clinical, laboratory, or imaging parameters. These findings should be interpreted with caution, and larger studies are warranted to determine whether these observed correlation coefficients represent true associations. Overall, no statistically robust correlations were identified in this analysis (Table 5).

**Figure 4:**
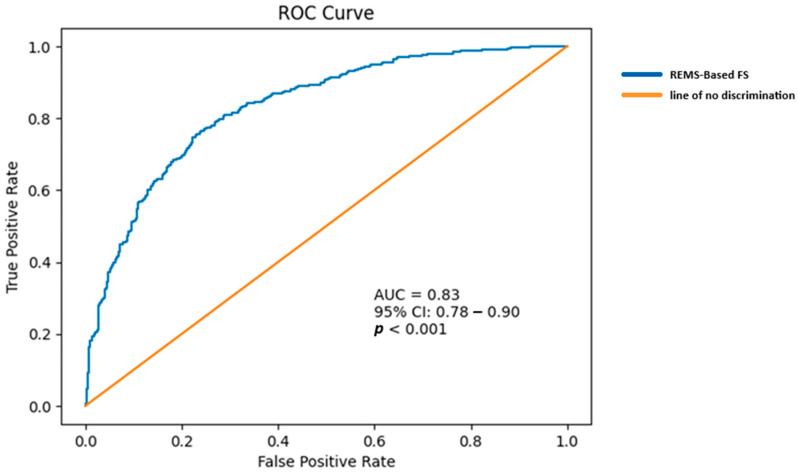
ROC Curve Analysis of REMS-Based FS for Differentiating R-axSpA Patients from Healthy Controls.

Receiver operating characteristic (ROC) curve analysis demonstrated that the REMS-derived Fragility Score (FS) showed good discriminative ability for distinguishing patients with r-axSpA from healthy controls, with an AUC of 0.83 (95% CI: 0.78–0.90, *p* < 0.001) and sensitivity of 82.2% and a specificity of 76.0% (Figure 4).

## 4. Discussion

Our study is the first to investigate surrogate markers of bone fragility in patients with r-axSpA using the REMS-based FS. The distribution of sex was comparable between both groups. However, the r-axSpA group was significantly younger than the control group (*p* < 0.001) and body mass index was also significantly lower in the r-axSpA group (*p* < 0.001). These baseline differences should be considered when interpreting the study findings, as they may have contributed to residual confounding.

Patients with r-axSpA showed significantly elevated serum C-reactive protein (CRP) levels (*p* < 0.001) as well as increased erythrocyte sedimentation rate (ESR) (*p* < 0.001). These findings are consistent with those reported by Korczowska et al., who also demonstrated increased inflammatory markers in patients with axSpA [20]. Furthermore, we observed a higher prevalence of smokers in the axSpA group compared to the control group (*p* < 0.001). In combination with the elevated inflammatory parameters, our results are in line with those of Zhang et al., who investigated disease activity in patients with ankylosing spondylitis and demonstrated an association between smoking and increased ESR [21].

Most patients with r-axSpA received nonsteroidal anti-inflammatory drugs (NSAIDs). A substantial proportion of patients were treated with bDMARDs, including TNF inhibitors in 36 patients (40%) and IL-17 inhibitors in 9 patients (10%). csDMARDs were used in 28 patients (31%), while systemic glucocorticoid therapy was reported in 14 patients (15%). Driscoll et al. investigated 2026 patients with axSpA in terms of fracture risk. In their cohort, TNF inhibitors were used in 34% of patients, csDMARDs in approximately 9%, and NSAIDs in 16%, while 40% received no treatment [22]. Regarding TNF inhibitor use, our cohort shows a similar proportion, whereas NSAID use was considerably higher in our study population.

Our study found no relevant differences in T-score or BMD between the axSpA and control groups (*p* = 0.8 and *p* = 0.6, respectively). Kim et al. reported reduced BMD in patients with axSpA [23]. Although the younger age of the r-axSpA cohort may have influenced these findings, the observed differences between the study groups may also reflect residual confounding resulting from incomplete baseline comparability. Therefore, the absence of significant differences in BMD and T-score should be interpreted with caution. Meirelles et al. investigated the impact of disease activity in ankylosing spondylitis and demonstrated that, when age was comparable between groups, patients with axSpA had significantly lower BMD than age-matched controls [24]. These findings highlight that age has a major influence on bone mineral density, even in chronic autoinflammatory diseases.

Regarding fracture risk, we demonstrated a significant difference between the r-axSpA group and the control group (*p* = 0.004). Kang et al. assessed fracture risk in axSpA patients using DXA and FRAX and confirmed an increased fracture risk in this population. They also reported reduced bone mineral density in axSpA patients [25]. These findings are consistent with our results in terms of increased fracture risk. Importantly, we were able to demonstrate for the first time that, despite comparable bone quantity, bone quality appears to be markedly reduced in patients with r-axSpA. This is reflected in increased skeletal fragility. The REMS-based fracture risk assessment therefore appears to provide a clear advantage over the radiography-based DXA method and the FRAX risk calculator.

Within the r-axSpA group, no statistically significant correlations were identified between REMS-derived fracture risk and disease activity or functional parameters, including ASDAS-CRP, BASFI and BASMI. Although correlation coefficients of moderate magnitude were observed for some variables, none reached statistical significance and therefore no conclusions regarding an association between disease activity, functional impairment and skeletal fragility can be drawn from the present data. The absence of statistically significant correlations may be related to the limited sample size and should be investigated in larger prospective studies. Our findings are consistent with those of Meirelles et al., who also reported no significant association between disease activity and inactivity, while demonstrating significant differences between axSpA patients and controls [24].

### Strengths and Limitations

A major strength of this study is the novel application of the REMS-derived FS for the cross-sectional assessment of skeletal fragility in patients with r-axSpA, enabling the evaluation of bone quality beyond conventional DXA-derived BMD. The inclusion of a control group and the comprehensive assessment of clinical, laboratory, and therapeutic variables further strengthen the validity of the study. Additionally, the study provides a detailed characterization of disease activity, functional status and inflammatory burden, enabling an integrated evaluation of potential determinants of skeletal fragility in r-axSpA.

However, several limitations should be acknowledged. The cross-sectional design precludes causal inferences regarding the relationship between disease-related factors and skeletal fragility. The sample size may have limited the statistical power to detect significant correlations between FS and clinical parameters. Furthermore, although the study groups were selected to improve comparability, significant baseline differences remained, particularly in age, body mass index and baseline bone characteristics. Consequently, residual confounding cannot be excluded and may have influenced the observed associations. This limitation should be considered when interpreting the comparative analyses. Moreover, mSASSS was not available for our cohort, preventing objective quantification of structural spinal damage and precluding assessment of its potential relationship with the REMS-derived findings. Future studies incorporating validated radiographic scoring systems, such as mSASSS, are needed to further investigate the association between structural disease severity and REMS-derived skeletal fragility. Finally, longitudinal data are lacking and future prospective studies are required to evaluate the predictive value of REMS-derived FS for incident fractures in r-axSpA.

## 5. Conclusions

In this study, patients with r-axSpA demonstrated comparable REMS-based bone mineral density and REMS-based T-scores to healthy controls; however, REMS-derived FS identified significantly increased skeletal fragility in the r-axSpA group. These findings suggest that conventional DXA-based bone assessment may underestimate skeletal deterioration in r-axSpA, as it primarily reflects bone quantity rather than bone quality. REMS therefore appears to provide additional clinically relevant information by providing a surrogate assessment of bone fragility beyond conventional DXA. No statistically significant correlations were identified between REMS-derived skeletal fragility and disease activity parameters. Overall, REMS-based assessment may represent a valuable complementary tool for the cross-sectional evaluation of skeletal fragility in patients with r-axSpA.

## Figures and Tables

**Table 1:** Baseline demographic, clinical and laboratory characteristics of patients with radiographic axial spondyloarthritis and controls.

Variable	r-axSpA (*n* = 90)	Controls (*n* = 100)	*p*-Value
Age (years)	46 ± 5	53 ± 4	<0.001
Female sex, *n* (%)	54 (60%)	64 (64%)	0.803
BMI (kg/m^2^)	26.1 ± 3.5	31.5 ± 5.9	<0.001
Disease duration (years)	7.5 ± 4.0	—	—
HLA-B27 positivity, *n* (%)	76 (84%)	—	—
Current smoking, *n* (%)	54 (60%)	30 (30%)	<0.001
Previous fracture, *n* (%)	36 (40%)	20 (20%)	0.01
Postmenopausal women, *n* (%)	40 (44%)	80 (80%)	0.01
CRP (mg/L)	10 ± 12	2 ± 1.5	<0.001
ESR (mm/h)	25 ± 18	8 ± 6	<0.001
BASDAI	4.8 ± 2.0	—	—
ASDAS-CRP	3.0 ± 1.0	—	—
BASFI	4.2 ± 2.1	—	—
BASMI	3.5 ± 1.8	—	—

**Table 2:** Multivariable linear regression analysis of factors associated with FS.

Variable	β (SE)	Standardized β	95% CI	*p*-Value
r-axSpA (vs. Control)	0.39 (0.09)	0.38	0.21 to 0.57	<0.001
Age (years)	0.011 (0.004)	0.20	0.003 to 0.019	0.006
BMI (kg/m^2^)	0.004 (0.007)	0.04	−0.010 to 0.018	0.576
Female sex	−0.028 (0.074)	−0.03	−0.174 to 0.118	0.705
Current smoking	0.118 (0.055)	0.16	0.010 to 0.226	0.032
Previous fracture	0.143 (0.061)	0.18	0.023 to 0.263	0.020
Postmenopausal status	0.052 (0.068)	0.06	−0.082 to 0.186	0.446
CRP (mg/L)	0.013 (0.004)	0.24	0.005 to 0.021	0.002
ESR (mm/h)	0.004 (0.002)	0.15	0.001 to 0.008	0.018
Model statistics				
Number of participants	190			
R^2^	0.59			
Adjusted R^2^	0.57			
F statistic	29.8			
Overall model *p*-value	<0.001			

**Table 3:** Pharmacological treatment and supplementation patterns in patients with r-axSpA.

Variable	*n* (%)
NSAID use	68 (75%)
TNF inhibitor therapy	36 (40%)
IL-17 inhibitor therapy	9 (10%)
Conventional DMARDs	28 (31%)
Glucocorticoids	14 (15%)
Calcium supplementation	45 (50%)
Vitamin D supplementation	59 (65%)
Anti-osteoporotic treatment	14 (15%)

**Table 4:** REMS-derived bone mineral density, T-scores, and fragility score comparing patients with radiographic axial spondyloarthritis and controls.

Parameter	r-axSpA	Controls	*p*-Value
Lumbar spine REMS-based BMD (g/cm^2^)	0.810	0.821	0.6
Lumbar spine REMS-based T-score (SD)	−1.4	−1.3	0.8
REMS-based Fragility Score	46.6 ± 15.4	31.2 ± 13.3	0.004
Risk Category	R5	R4	—
Femoral neck REMS-based BMD (g/cm^2^)	0.735	0.760	0.3
Femoral neck REMS-based T-score	−1.5	−1.3	0.6
Total hip REMS-based BMD (g/cm^2^)	0.830	0.850	0.2
Total hip REMS-based T-score (SD)	−1.1	−0.9	0.6

**Table 5:** Correlation analysis between clinical, inflammatory and bone parameters in patients with radiographic axial spondyloarthritis.

Variable	Correlation Coefficient (r)	*p*-Value
Age	0.40	0.14
Disease duration	0.32	0.23
BASDAI	0.25	0.37
ASDAS-CRP	0.38	0.15
CRP	0.28	0.30
BASFI	0.42	0.11
BASMI	0.48	0.06
Lumbar spine REMS-based BMD	0.32	0.24

## Data Availability

The authors confirm that the data supporting the findings of this study are not publicly available due to privacy and ethical restrictions.
